# ^1^H, ^13^C, and ^15^N backbone and side chain resonance assignments of the C-terminal DNA binding and dimerization domain of v-Myc

**DOI:** 10.1007/s12104-012-9437-3

**Published:** 2012-11-20

**Authors:** Gönül Kızılsavaş, Saurabh Saxena, Szymon Żerko, Wiktor Koźmiński, Klaus Bister, Robert Konrat

**Affiliations:** 1 Max F. Perutz Laboratories, Department of Structural and Computational Biology, University of Vienna, Vienna Biocenter Campus 5, 1030 Vienna, Austria; 2 Faculty of Chemistry, University of Warsaw, Pasteura 1, 02-093 Warsaw, Poland; 3 Institute of Biochemistry, Center for Molecular Biosciences (CMBI), University of Innsbruck, Innrain 80-82, 6020 Innsbruck, Austria

**Keywords:** Myc, Oncogene, Transcription factor, Random sampling, Intrinsically disordered protein

## Abstract

The oncogenic transcription factor Myc is one of the most interesting members of the basic-helix-loop-helix-zipper (bHLHZip) protein family. Deregulation of Myc via gene amplification, chromosomal translocation or other mechanisms lead to tumorigenesis including Burkitt lymphoma, multiple myeloma, and many other malignancies. The oncogene *myc* is a highly potent transforming gene and capable to transform various cell types in vivo and in vitro. Its oncogenic activity initialized by deregulated expression leads to a shift of the equilibrium in the Myc/Max/Mad network towards Myc/Max complexes. The Myc/Max heterodimerization is a prerequisite for transcriptional functionality of Myc. Primarily, we are focusing on the apo-state of the C-terminal domain of v-Myc, the retroviral homolog of human c-Myc. Based on multi-dimensional NMR measurements v-Myc appears to be neither a fully structured nor a completely unstructured protein. The bHLHZip domain of v-Myc does not exist as a random coil but exhibits partially pre-formed α-helical regions in its apo-state. In order to elucidate the structural propensities of Myc in more detail, the backbone and side-chain assignments obtained here for apo-Myc are a crucial prerequisite for further NMR measurements.

## Biological context

The v-*myc* oncogene encoding the v-Myc protein is the transforming principle of avian Myelocytomatosis virus MC29 (Bister and Jansen 1986), an acute leukemia virus isolated from a chicken tumor (Ivanov et al. 1964). The viral genome encodes the transforming protein in form of a Gag-Myc hybrid protein encompassing 450 N-terminal residues encoded by the 5′ end of *gag*, linked to nine residues encoded by sequences derived from noncoding regions of c-*myc* (cellular *myc*), followed by 416 residues encoded by sequences derived from the protein coding regions of exons 2 and 3 of c-*myc* (Reddy et al. 1983; Bister and Jansen 1986). The v-Myc amino acid sequence differs from that of c-Myc by only a few amino acid substitutions. There are multiple mechanisms to drive c-*myc* into deregulated expression such as retroviral transduction, viral integration, chromosomal translocation, and gene amplification. Alteration of human c-*myc* via chromosomal translocation was found in Burkitt lymphoma (Dalla-Favera et al. 1982). Translocation of c-*myc* is also frequently seen in multiple myeloma cases (Shou et al. 2000). An enhanced TCF transcriptional activation of c-*myc* can be induced by defects in the Wnt-APC pathway (He et al. 1998). The transduced v-*myc* allele is one of the most potent transforming genes (Bister and Jansen 1986; Frykberg et al. 1987). It is capable to transform several different lineages of mammalian and avian cells. The most prominent fate for a cell after transformation by v-*myc* is enhanced proliferation. A v-*myc* transformed cell can become growth factor independent and as a consequence can develop an immortal and tumorigenic phenotype.

Myc’s importance and power over the fate of cells derives from the fact that Myc is a transcription factor. Together with its C-terminal binding partner Max (Myc associated factor X) it binds as a heterodimer to its cognate E-box DNA sequence (5′-CACGTG-3′) found frequently throughout the genome. A large number of target genes have been found. Currently the Myc database (myccancergene.org) lists more than 1,600 target genes. The Myc target genes are involved in many different functional processes including cell cycle control, cell growth, metabolism, apoptosis and immortality. The bHLHZip domain of Myc is essential for binding to Max and ranges from residue 314 to 416 of full-length v-Myc. Structural preformation of Myc in the apo-state (prior to Max binding) has already been shown via multi-dimensional NMR (Fieber et al. 2001). The partially folded monomeric Myc showed dynamic features such as helical fraying and a directed flexibility in the loop region.

Myc is thus another example for the growing evidence that binding interactions with intrinsically disordered proteins (IDP) largely proceed via conformational selection from a significantly narrowed conformation ensemble. In order to properly characterize the preformed structural ensemble of Myc, a complete signal assignment including side-chains is indispensible. Severe spectral overlaps make complete signal assignment a challenging task in IDPs.

Lavigne et al. provided 1998 a solution structure of N-terminal disulfide-linked versions of the c-Myc-Max heterodimeric Leucine Zipper solved by 2D H-NMR. A full backbone assignment of v-Myc in complex with its binding partner Max was published by Baminger et al. in 2004. Here we provide a complete signal assignment, backbone and side-chain, for the bHLHZip domain of apo (monomeric) v-Myc, using a set of high-dimensional triple resonance NMR experiments. Apo v-Myc shows a different spectra in respect to the heterodimeric Myc-Max complex. For more details compare with BMRB entry 6163. Our data will not only be crucial for the characterization of Myc’s apo-state, but also a valuable source of information for the analysis of amino-acid side chains in IDPs.

## Methods and results

### Expression and purification of v-Myc

The v-Myc bHLHZip domain encoding fragment was obtained by cleavage of the pET3dmycmax expression plasmid (Fieber et al. 2001; Baminger et al. 2004) using BamH1 and NcoI. The resulting fragment was ligated into the pETM-11 expression vector providing v-Myc with a N-terminal His-tag. The pETM-11v-myc plasmid was transformed into Rosetta(DE3)pLysS cells for protein expression following a protocol for efficient isotopic labeling of recombinant proteins using a fourfold cell concentration in minimal medium containing ^15^NH_4_Cl and ^13^C-glucose (Marley et al. 2001). v-Myc was expressed in inclusion bodies. The cells were collected after overnight expression at 30 °C by centrifugation at 4,000 rpm for 15 min and resuspended in 30 ml of ice-cold lysis buffer (20 mM NaH_2_PO4/Na_2_HPO4, 100 mM NaCl, 1 mM EDTA, 1 mM DTT, pH 6.5). The cells were lysed via sonication at 70 % for 3 × 5 min. After cell lysis, the lysate was cleared by centrifugation at 18,000 rpm for 20 min. The pellet was resuspended in 8 M Urea (including 1 mM EDTA, 100 mM NaCl and 20 mM imidazole, pH 8) and loaded onto a Ni^2+^-loaded HisTrap FF 5 ml affinity column (GE Healthcare) and eluted with 8 M Urea containing 100 mM imidazole. The eluate was collected and a step-wise refolding in 4 steps with buffer (20 mM NaH_2_PO4/Na_2_HPO4, 100 mM NaCl, 1 mM EDTA, 1 mM DTT, pH 6.5) was performed at 4 °C. After refolding the sample was concentrated to approximately 0.7 mM.

### NMR experiments

All spectra were acquired at 298 K on an Agilent Direct Drive 700 MHz spectrometer using a standard 5 mm ^1^H–^13^C–^15^N triple-resonance probehead.

The backbone ^1^H, ^13^C, and ^15^N resonances were assigned using sparse random sampling of indirectly detected time domains, in order to increase resolution. A 3D HNCO experiment was used as a base spectrum for SMFT (Sparse Multidimensional Fourier Transform) processing of higher dimensionality experiments (Kazimierczuk et al. 2010). Backbone assignment was achieved using 5D HN(CA)CONH (Kazimierczuk et al. 2010), (HACA)CON(CA)CONH, (H)NCO(NCA)CONH and HNCOCACB (Zawadzka-Kazimierczuk et al. 2012b) experiments. Side-chain assignments were obtained using 5D HabCabCONH (Kazimierczuk et al. 2010), and H(CC-tocsy)CONH (Kazimierczuk et al. 2009) experiments.

All NMR data sets were processed by multidimensional Fourier transformation using the home written software package (http://nmr700.chem.uw.edu.pl). The resonance assignment was performed using the TSAR program (Zawadzka-Kazimierczuk et al. 2012a). The input data for TSAR was prepared using Sparky software (Goddard and Kneller 2002).

### Extent of assignment and data deposition

**Fig. 1:**
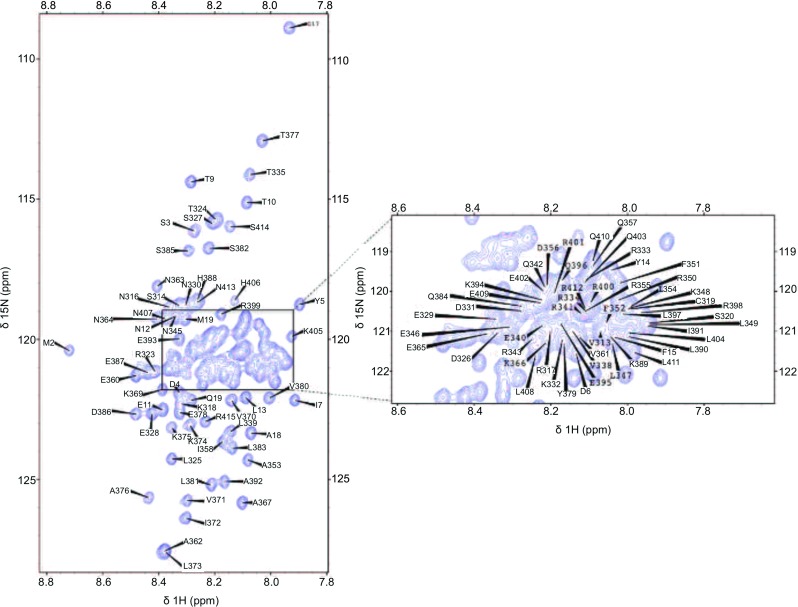
2D ^15^N-HSQC spectrum with a zoom of the central crowded region of his-tagged v-Myc at pH 6.5 and 25 °C. Assignments of backbone amides are indicated with the one-letter code for amino acids and the residue numbers (his-tag:1-27, v-Myc:314-416)

**Fig. 2:**
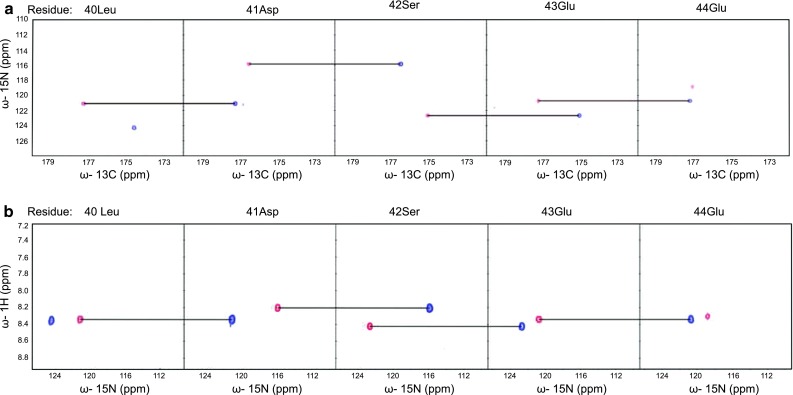
2D spectral planes for v-Myc obtained by SMFT procedure performed on the 5D randomly sampled signal (Poisson disk sampling). **a** (HACA)CON(CA)CONH and **b** HN(CA)CONH

The ^1^H–^15^N HSQC spectrum of v-Myc shows a very narrow peak dispersion in the ^1^H dimension typical for intrinsically disordered proteins (Fig. 1). Extensive signal overlap in conventional 2D and 3D spectra could be overcome by using the aforementioned 5D experiments. 97.1 % of backbone ^15^N, 96 % of ^1^H^N^, 91.3 % of ^13^C^α^, 90.4 % of ^1^H^α^, 91.3 % of ^13^C^β^, 90.4 % of ^1^H^β^ and 95.2 % of ^13^C′ resonances have been assigned (calculated without His-tag). Figure 2 shows sample strips of sequential resonance assignment in a 5D (HACA)CON(CA)CONH and HN(CA)CONH experiment. Additionally, H(CC-tocsy)CONH spectra allowed the assignment of several side-chain atoms. 43 % of C^γ^ (calculated without His), 50 % of H^γ^, 32 % of C^δ^, and 42.1 % of H^δ^ could be assigned.

The assignments have been deposited in the BioMagResBank database (http://www.bmrb.wisc.edu) under accession number: 18580.
